# End-of-life decision-making capacity in an elderly patient with schizophrenia and terminal cancer

**DOI:** 10.4102/safp.v62i1.5111

**Published:** 2020-08-03

**Authors:** Carla Kotze, Johannes L. Roos

**Affiliations:** 1 Department of Psychiatry, Weskoppies Psychiatric Hospital, University of Pretoria, Pretoria, South Africa

**Keywords:** end-of-life, decision-making capacity, older people, serious mental illness, Schizophrenia

## Abstract

Medical practitioners are confronted daily with decisions about patients’ capacity to consent to interventions. To address some of the pertinent issues with these assessments, the end-of-life decision-making capacity of a 72-year-old female with treatment-resistant schizophrenia and terminal cancer is discussed, as are the role of the treating clinician and the importance of health-related values. There is a recommendation that the focus of these assessments can rather be on practical outcomes, especially when capacity issues arise. This implies that the decision-making capacity of the patient is only practically important when the treatment team is willing to proceed against the patient’s wishes. This shifts the focus from a potentially difficult assessment to the simpler question of whether the patient’s capacity will change the treatment approach. Clinicians should attend to any possible underlying issues, instead of focusing strictly on capacity. Compared to the general populations people with serious mental illness (SMI) have higher rates of physical illness and die at a younger age, but they do not commonly access palliative care services. Conversations about end-of-life care can occur without fear that a person’s psychiatric symptoms or related vulnerabilities will undermine the process. More research about palliative care and advance care planning for people with SMI is needed. This is even more urgent in light of the coronavirus disease-2019 (COVID-19) pandemic, and South African health services should consider recommendations that advanced care planning should be routinely implemented. These recommendations should not only focus on the general population and should include patients with SMI.

## Introduction

Medical practitioners are confronted on a daily basis with decisions about patients’ capacity to consent to interventions as required by law and medical ethics. These processes can be challenging, even more so in vulnerable populations, such as older people with pre-existing serious mental illness (SMI). The patient should remain central in decision-making with consideration of the patient’s personal and cultural values to assure autonomous decision-making.^1^

There can be discrepancies between clinical and formal capacity assessments. Most medical practitioners will use their own subjective judgement and clinical experience in situations where capacity has to be assessed, but all such assessments should be done in the context of a thorough clinical evaluation.^2^ The ultimate goal of determining capacity is to maintain a proper balance between respect for patient autonomy and protecting those who lack capacity from making harmful decisions. Informed consent can only be considered valid if a competent person is permitted to make a voluntary choice after disclosure of appropriate information.^3^

The novel coronavirus disease 2019 (COVID-19) pandemic has made advance care planning prior to serious acute illness even more urgent, as there are potential needs for the rationing of healthcare in the context of scarce resources. This pandemic heightens the need for having discussions about goals of care, to avoid non-beneficial or unwanted interventions, especially in patients with chronic, life-limiting disease.^4^

To address some of the difficulties with these capacity assessments, the end-of-life decision-making capacity in a 72-year-old female with treatment-resistant schizophrenia and terminal cancer, will be discussed.

The diagnosis of schizophrenia was made when she was 16 years old. At school, she experienced socialisation difficulties and frequently complained that she felt alone and forsaken. This behavior affected her academic progress and she discontinued her education in grade 10, because of her illness. She frequently experienced depressive symptoms. She was single, with no children, and resided in an old-age home.

She was able to work in a sheltered employment position for 13 years, after which she was medically boarded. During her illness, she was hospitalised on several occasions before she followed up as a voluntary outpatient at a tertiary psychiatric hospital for approximately 40 years. Her clinical picture included fixed delusions and prominent negative symptoms of schizophrenia that impaired her functioning.

Towards the end of 2018, she presented gastrointestinal symptoms and weight loss. She was referred for further investigation, where she was diagnosed with an adenocarcinoma of her stomach with local invasion of the oesophagus. Her condition was considered terminal, as she had a progressive life-limiting disease with a prognosis of months or less.^5^ No active curative treatment was planned and she returned to her living circumstances, with treatment to manage her pain.

Before her cancer diagnosis, she was interviewed as part of a research endeavour to ascertain her decision-making capacity about end-of-life care by an independent psychiatrist. With this assessment, she was found not to have decision-making capacity and this is compared to the assessment done by her treating psychiatrist after she was diagnosed with cancer. Her treating psychiatrist was of the opinion that she did have decision-making capacity, highlighting some of the difficulties with these assessments. These types of decision-making assessments can be complicated by the deficits originating from the neurodevelopmental aspects and the related cognitive impairments of schizophrenia, even more than the psychotic symptomatology of the illness.^6^

The following areas of importance in daily clinical care of patients with schizophrenia and other SMIs will be discussed: health-related values and capacity assessment; the role of the treating clinician; the pragmatic approach; and end-of-life care preferences in persons with SMI.

## Health-related values and capacity assessment

Many psychiatric disorders are associated with impaired capacity, but there is a great variation between different disorders.^2^ Acute episodes in schizophrenia or mania in bipolar disorder have a much stronger association with impaired decision-making capacity than major depressive disorder.^7^ However, lack of insight has been reported as the strongest predictor of incapacity and the assignment of specific diagnostic categories should not be confused with termination of capacity.^3^ There is no specific condition that precludes a patient’s ability to make a competent decision, and decision-making capacity is specific to a particular decision rather than to a diagnosis. Mental capacity is not associated with any socio-demographic variable apart from advancing age, but little is known about the effects of ageing on the capacity of patients with SMI.^8^

Concern exists regarding the reliability of capacity assessments in individuals with SMI. Capacity assessments can be complex and value-laden, but it can be assessed with fair reliability.^8^ Instruments have been developed to improve the subjectivity of capacity assessments, but normative data are lacking and currently, expert opinion is considered the ideal. Detection of incapacity for decision-making depends on an appropriate level of suspicion and can be improved by clarification of applicable criteria and the use of a systematic approach.^9^

This patient, as described in the introduction, participated in a study that investigated the end-of-life decision-making capacity in older people with SMI. An independent forensic psychiatrist performed an assessment using the legally-relevant criteria for decision-making capacity and approaches to assessment of the patients described by Grisso and Appelbaum (1998), which comprises four essential abilities. This model includes the ability to understand the pertinent information, to appreciate the circumstances and the consequences, to reason about the choices, and to communicate a choice. This is considered a standard for capacity assessments.^10^ After this assessment, the researcher administered the standardised Assessment of Capacity to Consent to Treatment (ACCT) interview. The ACCT begins with an interview to elicit values and preferences relevant to healthcare decision-making. After this, a vignette was read to the patient and she was also provided with a summary, to decrease reliance on memory. A structured questionnaire about the vignette was then administered.^1^

The patient performed poorly on a cognitive screening test, with a score of 1 out of 5 on the Mini-cog.^11^ The three values that she identified as being most important to her were being able to take care of herself, to have relationships with family and friends, and to live without significant pain or discomfort. She wanted to make shared health decisions with her doctor, but she did not want any family to be involved. Quality of life was more important than how long she lived and if she were very sick, she would not want anything done to prolong her life. In contrast with these preferences, she indicated that she would want cardiopulmonary resuscitation if her heart stops after she had a debilitating stroke. She did not display distrust, but had prominent cognitive impairments. The researcher was of the opinion that she did not have end-of-life decision-making capacity.

The involvement of family in advance care discussions and decisions is essential, and they should be made aware of the person’s health-related values and preferences. When a patient does not have family support available, as was the situation in this case, and in the absence of advance care planning documentation such as a living will, their health-related values can be especially important to guide treatment preferences.^12^

## The role of the treating clinician

As was found in this case, clinicians tend to be less likely to make a judgement of incapacity. In general, assessments by researchers have been found to be less reliable than capacity assessments performed by treating clinicians. It has been suggested that clinicians presume that capacity is present if a patient is prepared to accept the proposed treatment. In a routine consultation, there might also not be sufficient time for a careful assessment to be performed, but formal assessments can lack specificity and over-diagnose incapacity.^8^

The final judgement about capacity is a clinical judgement that should draw upon a wide variety of evidence, including the clinical interview, formal capacity measures, other cognitive tests and the experience of the evaluator. This should always be applied to a specific situation and be weighed against the possible risks associated with a particular decision.^3^ In this patient’s case, her prominent cognitive impairment and poor insight were the main concerns.

## The pragmatic approach

Palliative care is an approach that improves the quality of life of patients and their families facing the problems associated with life-threatening illness. In patients who have serious health-related suffering because of a life-limiting illness and whose condition is deteriorating and where it is likely that the patient’s death will be a consequence of the illness, palliative care should be integrated in all of their care. The aim should be to treat pain, manage symptoms, relieve suffering, provide psychosocial and spiritual support, and not to prolong or hasten death.^13^ Patients should be encouraged to take ownership of their illness and it is good clinical practice for both the clinician and patient to participate in the planning and choosing for the road ahead.^14^ However, stigma towards patients with SMI is prevalent and it often manifests in lower quality end-of-life care and the misattribution of medical symptoms to psychiatric illness.^15^

This case highlights the differences in opinion about decision-making capacity, but the end-of-life care and proposed treatment were not an issue here. It has been suggested that with such cases, the focus should be on the practical outcomes of the assessment. When capacity is unclear, an alternative is to rather check if the proposed treatment will be medically appropriate and available to be administered involuntarily, should a patient refuse. The capacity of the patient is only practically important when the treatment team is willing to proceed against the patient’s wishes. As in this case, the outcome of not treating her involuntarily would be the same as honouring her choice, and there is no need to assess capacity. Even in cases where a patient is found not to have decision-making capacity, this does not preclude the inclusion of the patient in treatment discussions or the consideration of her values in any decisions. The focus is just shifted to the simpler question of whether the patient’s capacity will change the treatment. By following this pragmatic approach and anticipating the outcomes of a capacity assessment, this situation can be transformed into an easier question. If treatment will not be administered involuntarily, whether or not the patient has capacity is immaterial to the question of what to do next. If this is the case, and prerequisites for opposing the patient’s choice are not present, there is no conflict that needs to be resolved.^16^

## End-of- life care preferences in people with serious mental illness

Most older people with SMI are able to engage in advance care planning and have decision-making capacity about their end-of-life care preferences. Conversations about end-of-life care can occur without fear that a person’s psychiatric symptoms or related vulnerabilities will undermine the process.^17^ Nevertheless, people with SMI are significantly less likely to access palliative care services, as the stigma associated with mental illness is associated with differences in access to healthcare compared to the general population.^18^

People with SMI bear a disproportionate burden of comorbid medical problems, and they often die at a younger age.^19^ Even so, these patients are rarely engaged in end-of-life care discussions and advance care planning, although it has been shown that they are receptive to these discussions. Clinicians often lack the knowledge and training needed to facilitate and support advance care planning in patients with SMI.^20^ People with SMI may not have family or friends available who can be substitute decision-makers and this, together with a presumption of incapacity and fear that end-of-life discussions will be emotionally and cognitively destabilising, also contributes to this problem.^19^

Clinicians are advised to attend to any possible underlying issues, instead of focusing strictly on capacity. The issues might include unattended needs, pain or discomfort, financial concerns or additional time to process information. Request for capacity assessments should not be based on underlying interpersonal or communication issues.^16^

The National Health Amendment Bill was enacted and published in the Government Gazette in March 2019. This amendment inserted sections 7A and 7B into Act 61 of 2003 to make provision for durable power of attorney for healthcare, and to give legal recognition and enforceability regarding living wills.^21^ Routine documentation of end-of-life care preferences can support future decision-making for family and clinicians at a time when patients are unable to express their decisions. There are limited data on the stability of patient preferences over time and capacity should not be viewed as an unmodifiable trait. Patients having difficulty with initial understanding of disclosed material can often benefit from educational efforts designed to teach them the relevant information.^20^ Advance care planning should be seen as a dynamic process, with regular revisions when clinical decisions need to be made.^22^

More research about palliative care and advance care planning for people with SMI is needed. This is even more urgent in light of the coronavirus disease 2019 (COVID-19) pandemic as well as the recent legal developments and advocacy efforts for enduring power of attorney and assisted suicide in South Africa. Health services should consider recommendations that advanced care planning should be implemented routinely. These recommendations should not only focus on the general population and should include patients with SMI.
